# Effects of a marine heatwave associated with the Kuroshio Extension large meander on extreme precipitation in September 2023

**DOI:** 10.1038/s41598-025-88294-9

**Published:** 2025-02-13

**Authors:** Hidetaka Hirata, Ryuichi Kawamura, Masami Nonaka

**Affiliations:** 1 Department of Data Science, Faculty of Data Science, Rissho University, Kumagaya, Japan; 2 Department of Earth and Planetary Sciences, Faculty of Science, Kyushu University, Fukuoka, Japan; 3 Application Laboratory, Japan Agency for Marine-Earth Science and Technology, Yokohama, Japan

**Keywords:** Climate sciences, Natural hazards

## Abstract

In 2023, the Kuroshio Extension (KE) exhibited an unusually large meander (LM) path, which caused a significant increase in sea surface temperatures, a marine heatwave (MHW), to the east of Japan. Although this MHW has the potential to influence heavy precipitation, it has not yet been examined. Here, we address this issue by examining an extreme precipitation event that occurred in Japan in September 2023 using high-resolution cloud-resolving numerical experiments. Results indicated that the MHW contributed to an increase in surface precipitation by ~ 300 mm, accounting for ~ 70% of the total precipitation. This precipitation intensification was attributed to the MHW-enhanced atmospheric instability and its influence on the formation and positioning of surface fronts. This research suggests that the KE-LM-induced MHW has elevated the risk of heavy precipitation in Japan, with significant societal implications. These findings could be applied to other regions such as the Northeastern United States.

**Supplementary Information:**

The online version contains supplementary material available at 10.1038/s41598-025-88294-9.

## Introduction

**Fig. 1:**
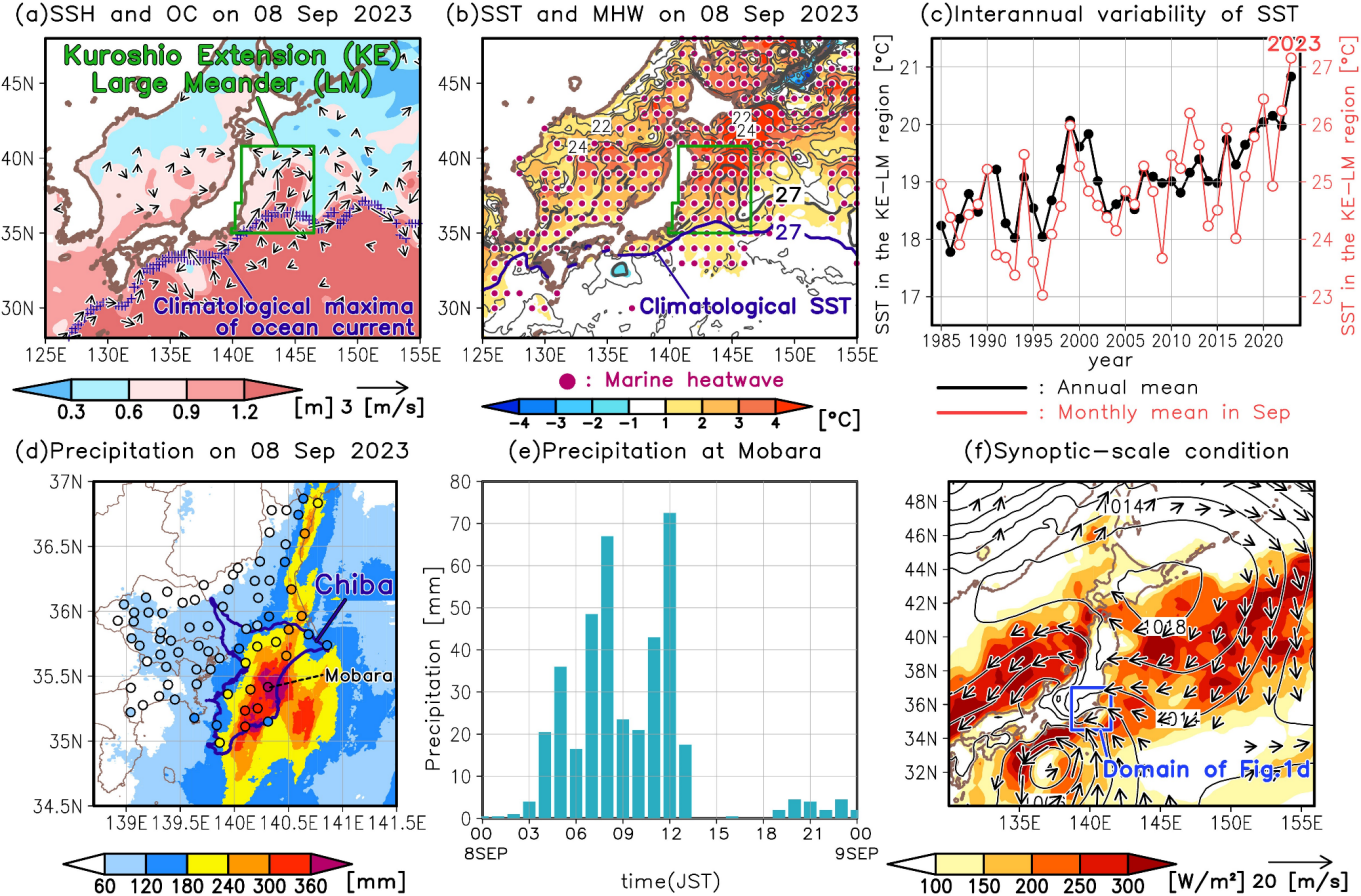
Characteristics of the marine heatwave associated with the Kuroshio Extension large meander and extreme precipitation in September 2023. (**a**) Horizontal distribution of sea surface height (shading) and surface geostrophic ocean current vectors (arrows) based on satellite observations on September 8, 2023. Blue crosses plot the maximum of the climatology (defined by the average from 1993 to 2023) of the magnitude of surface geostrophic ocean current on 8 September at each longitude. (**b**) Horizontal distribution of sea surface temperature (SST, gray contours) and its anomaly (shading) on September 8, 2023. Data used are from the Coral Reef Watch v3.1 SST product. Purple dots indicate the area where marine heatwaves occurred on that day. Gray contour interval is 1 °C. A dark blue line indicates the 27 °C contour of the climatology (defined by the average from 1985 to 2012) of SST on 8 September. (**c**) Time series of SST averaged over the oceanic area enclosed by the green line in (**a–b**). Black and red lines display the annual mean and the monthly mean for September, respectively. (**d**) Horizontal distribution of surface daily precipitation (shading) based on Radar/Raingauge-Analyzed Precipitation data produced by the Japan Meteorological Agency (JMA) on September 8, 2023. Colors of the circles indicate daily precipitation observed on that day at rain gauges of the JMA. (**e**) Time series of hourly precipitation at the Mobara station on September 8, 2023. Japan Standard Time (JST) is 9 h ahead of Universal Time Coordinated (UTC). (**f**) Horizontal distribution of heat fluxes from ocean to atmosphere (sum of sensible and latent heat fluxes, shading), sea level pressure (SLP, contours), and horizontal wind vectors at 10 m altitude (arrows) averaged from 00:00–13:00 JST on September 8, 2023. Data used are from ERA5. The contour interval is 4 hPa. A blue rectangle represents the domain of Fig. 1d.

Does a recent marine heatwave (MHW) induced by the large meandering (LM) of the Kuroshio Extension (KE) influence the occurrence of heavy precipitation? Since 2019, the KE has been meandering northward from its typical position to the east of Japan, and this pattern has remained quasi-stable^1,2^. This stable meandering of the KE first emerged in the period after 1993, when satellite measurements of sea surface height (SSH) began^1,2^, and it is continuing as of this writing (December 2024). In 2023, the sea surface temperature (SST) warming associated with the LM of the KE reached an extreme magnitude^3,4^ (Fig. 1a–c). Consequently, a MHW^5^ was observed in the region affected by the KE-LM^4^ (Fig. 1b). Given that SST variability in mid-latitudes is known to influence heavy precipitation^6–10^, it is hypothesized that the MHW associated with the KE-LM impacts the occurrence of heavy precipitation. However, the specific impact of the KE-LM has not yet been investigated.

This research examines the influence of the MHW induced by the KE-LM on heavy precipitation, focusing on the record-breaking precipitation event in Chiba Prefecture, Japan, on September 8, 2023 (Fig. 1d). On that day, the precipitation exceeding 100 mm/day was recorded at all of Japan Meteorological Agency (JMA) stations in Chiba Prefecture (Fig. 1d). Notably, the Mobara station in eastern Chiba Prefecture recorded precipitation of 391 mm/day, which is the highest daily precipitation since records started in January 1976 (Fig. 1d, e). This extreme precipitation led to flooding and landslides, causing significant damage.

The synoptic-scale atmospheric conditions on September 8, 2023, suggest that the MHW influenced this heavy precipitation event (Fig. 1f). At that time, Typhoon No. 13 was located southwest of Chiba Prefecture, and an anticyclone was located northeast of the main island of Japan. These conditions generated prevailing easterly winds around Chiba Prefecture. Around the MHW region upstream of eastern Chiba prefecture, heat fluxes from the ocean to atmosphere were observed. These atmospheric and oceanic conditions suggest that the MHW associated with the LM of the KE was responsible for the record-breaking heavy precipitation in eastern Chiba Prefecture. For these reasons, this study focuses on this case.

This study aims to clarify the influence of the MHW associated with the KE-LM on the extreme precipitation observed in September 2023. To achieve this, a control simulation experiment (CTL run) and sensitivity experiments to SST anomalies related to the MHW were conducted using a regional cloud-resolving numerical model^11^ with a horizontal grid spacing of 0.02°. The data obtained from these experiments were analyzed to assess the impact of the MHW on the heavy precipitation.

## Results

### Verification of reproducibility of the extreme precipitation in the simulation

In this section, we assess the reproducibility of precipitation in the CTL run by comparing the simulated data with observations from the JMA. We specifically examine the accumulated precipitation from 00:00 to 13:00 Japan Standard Time (JST) on September 8, 2023, during which intense precipitation was recorded at Mobara (refer to Fig. 1e).

**Fig. 2:**
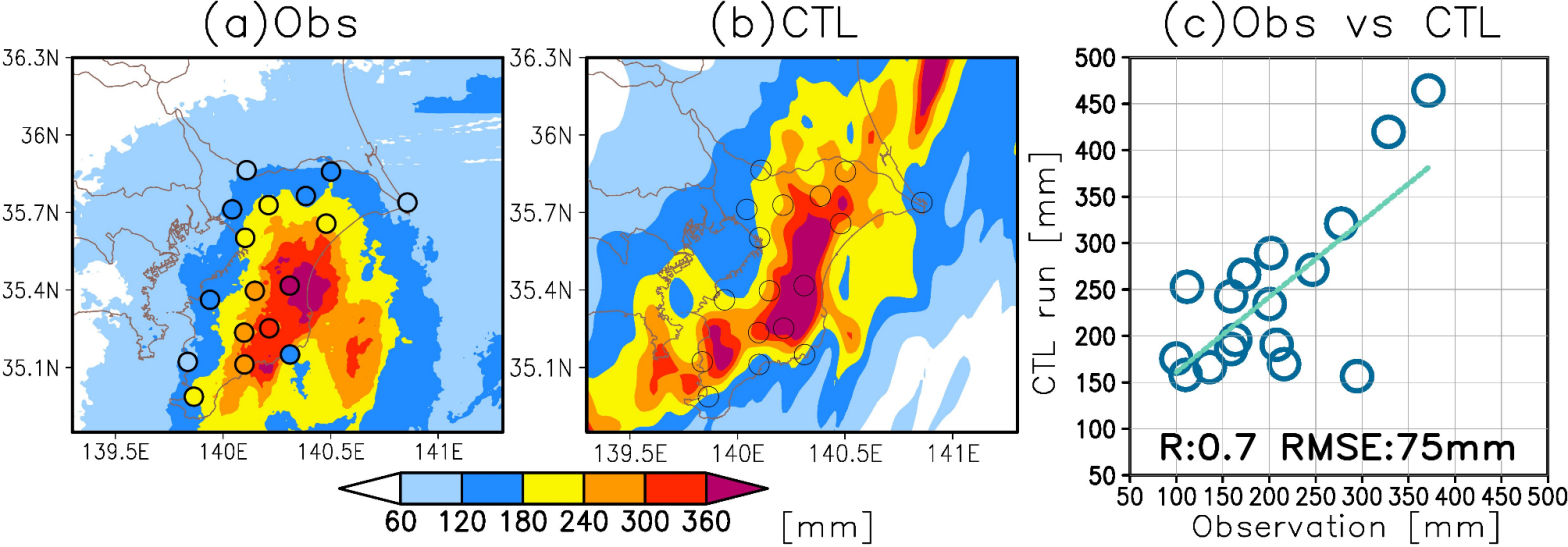
Comparison of precipitation between observations and the control run. (**a**) Surface precipitation accumulated from 00:00–13:00 JST on September 8, 2023. Shading indicates Radar/Raingauge-Analyzed Precipitation data, and colored circles indicates in-situ observations in Chiba Prefecture. (**b**) Same as (**a**), but the shading indicates the accumulated precipitation in the control (CTL) run. Open circles indicate the location of the observations. **c** Scatter plots between the rain-gauge observations in Chiba Prefecture and precipitation on the grids most proximate to those in the CTL run.

We analyze the spatial distribution of precipitation by comparing the Radar/Raingauge-Analyzed Precipitation data from the JMA with the CTL run output (Fig. 2a, b). The CTL run accurately captures the locally enhanced precipitation in the eastern part of Chiba Prefecture on September 8, 2023. However, the simulated precipitation in the CTL run is overestimated and shifted slightly northward compared to the observed data.

Furthermore, we compare the precipitation recorded by JMA rain gauges in Chiba Prefecture (indicated by closed circles in Fig. 2a) with that at the corresponding grid points in the CTL run (Fig. 2c). The correlation coefficient between the observed and simulated data is + 0.7, which suggests a consistent general agreement in the precipitation patterns in Chiba Prefecture. Nevertheless, the root mean square error is 75 mm, and the CTL run tends to overestimate precipitation compared to the observation. This bias may be due to the uncertainties associated with the model resolution, physical process parameterizations, and initial conditions.

These results suggest that while the CTL run tends to overestimate precipitation, it successfully reproduces the characteristics of local precipitation intensification in eastern Chiba Prefecture on September 8, 2023. Thus, we used the CTL run as the benchmark in this study. In the subsequent section, we will explore the precipitation response to the KE-LM-associated MHW by comparing the CTL run with an SST sensitivity experiment.

### Response of the extreme precipitation to the marine heatwave

In this section, we investigate the impact of the KE-LM-associated MHW on precipitation by comparing the CTL run with a numerical experiment where the SST in the KE-LM region (indicated by a green line in Fig. 1b) is replaced with climatological values (CLM run). The SST difference between the CTL and CLM runs exceeds 2 °C across a broad area within the KE-LM region, and notably more than 4 °C in the northern part of this region (refer to Fig. 1b, Supplementary Fig. 1).

**Fig. 3:**
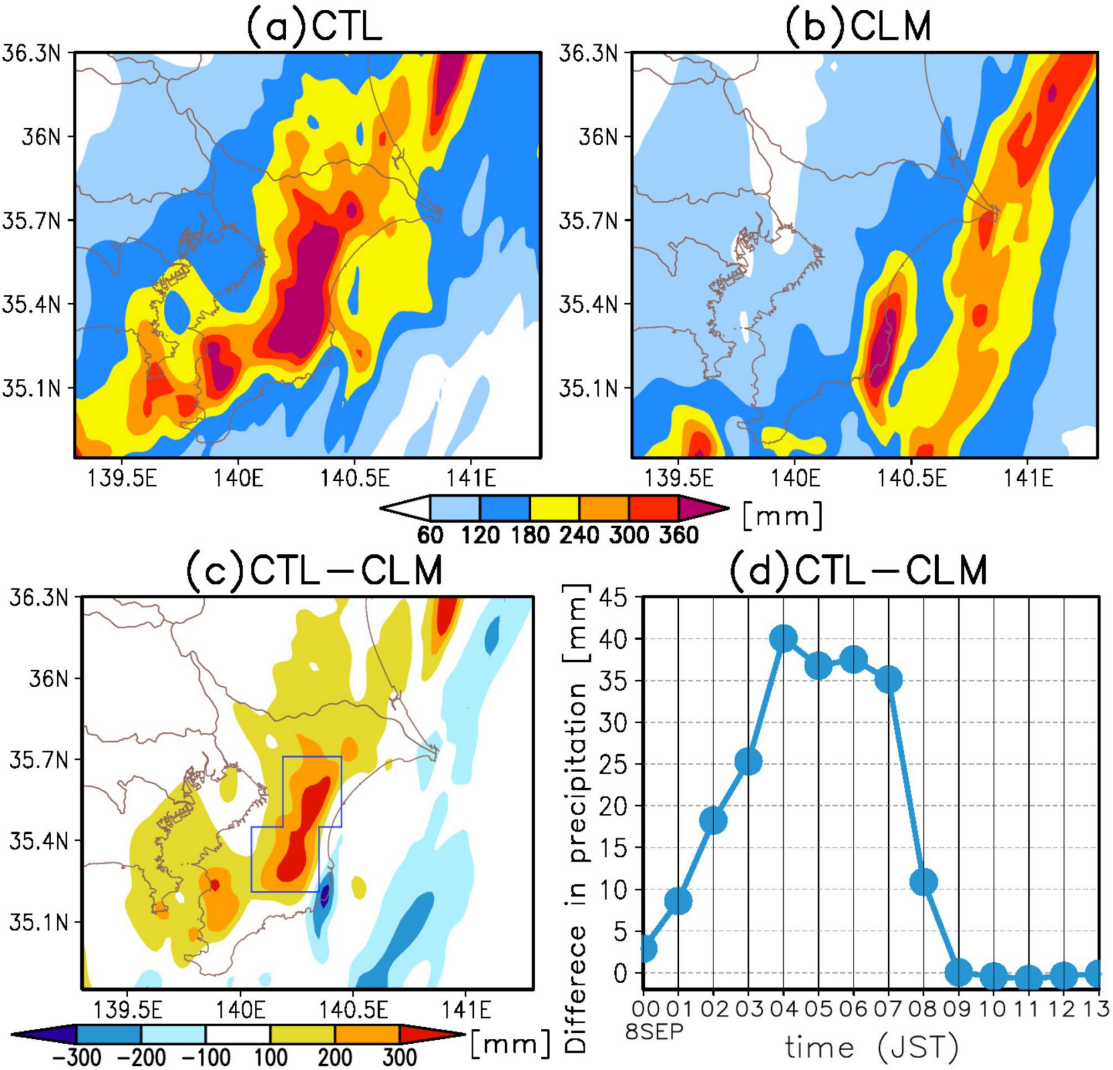
Response of precipitation to the marine heatwave. (**a**) Same as Fig. 2a. (**b**) Same as (**a**), but for the climate (CLM) run. (**c**) Horizontal distribution of the difference in surface precipitation accumulated from 00:00–13:00 JST on September 8, 2023, between the CTL and CLM runs. (**d**) Time series of the difference in hourly precipitation between the two runs averaged over the area enclosed the blue line in (**c**) (CTL run minus CLM run).

Figure 3a–c displays the spatial distribution of precipitation in the CTL and CLM runs and the differences between them. A significant reduction in precipitation is observed in the eastern part of Chiba Prefecture, where the intense precipitation was recorded in both the observation and the CTL run. The reduction exceeds 300 mm, approximately 70% of the precipitation recorded in the CTL run. Compared to the CTL run, the precipitation in the CLM run not only reduces, but also tends to shift eastward. Consistent with this, as illustrated in Supplementary Fig. 2, a linear regression analysis of data from 11 experiments, including the CTL and CLM runs, which differ only in the magnitude of SST anomalies within the KE-LM region, reveals a statistically significant positive regression coefficient of precipitation on SST anomalies in eastern Chiba Prefecture.

Figure 3d presents the time series of the difference in hourly precipitation between the CTL and CLM runs averaged over the area delineated by the blue line in Fig. 3c from 00:00–13:00 JST on September 8, 2023. This area corresponds to where the largest differences in precipitation between the two runs are observed. The difference starts to increase from 01:00 JST, peaking at 04:00 JST, remains elevated from 05:00–07:00 JST, and subsequently starts to decline from 08:00 JST. The temporal evolution of this difference will be further investigated.

### Effects of the marine heatwave on atmospheric conditions prior to the extreme precipitation

Earlier, we discussed the influence of the KE-LM associated MHW on the precipitation enhancement over eastern Chiba Prefecture. In subsequent sections, we explore how the MHW influenced this enhancement by comparing the CTL and CLM runs. The MHW may have created conditions conducive to precipitation enhancement by affecting the atmospheric conditions before the heavy precipitation event. Thus, this section focuses on the period just before the onset of precipitation (12:00–23:00 JST on September 7, 2023). The ensuing section will examine the atmospheric conditions during the heavy precipitation event (01:00–08:00 JST on September 8, 2023).

**Fig. 4:**
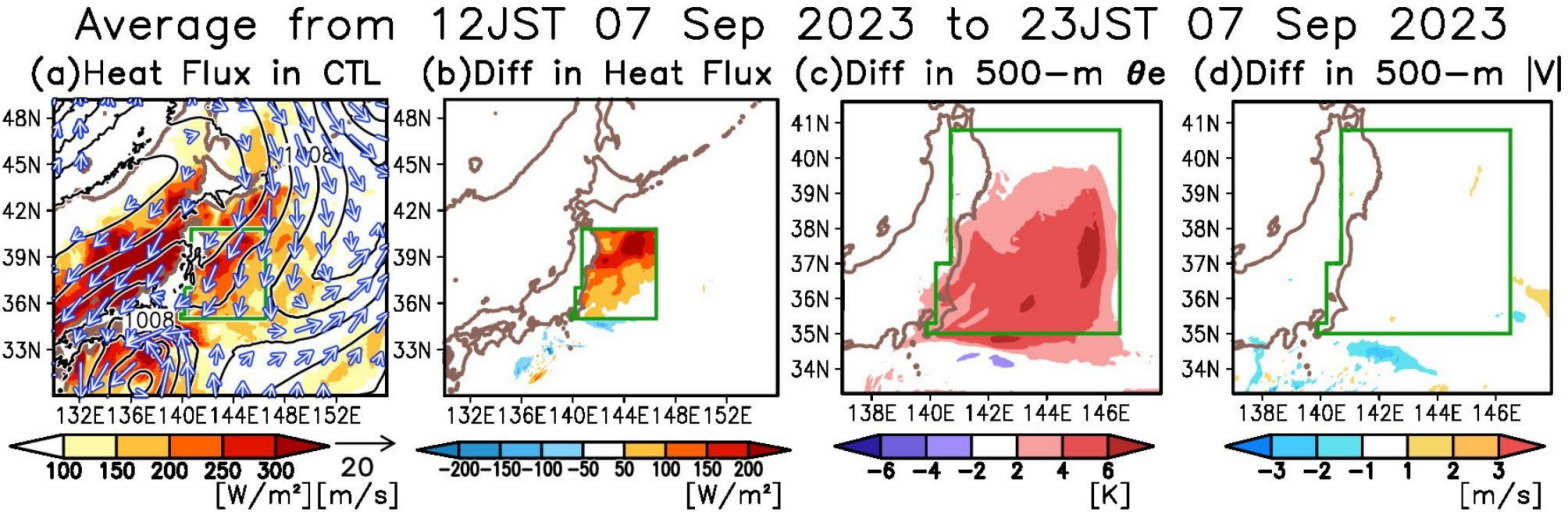
Effects of the marine heatwave on atmospheric conditions prior to the extreme precipitation. (**a**) Horizontal distributions of surface heat fluxes (sum of sensible and latent heat fluxes, shading), SLP (contours), and horizontal wind vector at 10 m altitude (arrows) averaged from 12:00–23:00 JST on September 7, 2023, in the CTL run. Contour interval is 2 hPa. Ocean area enclosed by the green line corresponds to the area where the SST is modified in the CLM run. (**b**) Horizontal distribution of the difference between the CTL and CLM runs for the surface heat flux averaged from 12:00–23:00 JST on 7 September 2023. (**c–d**) Same as (**b**), but the shading indicates the differences in equivalent potential temperature ($\:{\theta\:}_{e}$) at 500 m altitude and in the horizontal wind speed at 500 m altitude, respectively.

Initially, we analyzed the atmospheric conditions near the surface and the oceanic surface heat fluxes in the CTL run (Fig. 4a). Prior to the heavy precipitation event, a low-pressure system was present northeast of Japan, accompanied by prevailing northerly winds over the KE-LM region. These winds transported cold and dry air from higher latitudes to the KE-LM region, leading to a noticeable increase in surface heat flux (≥ 150 W/m^2^, Fig. 4a). It is likely that the MHW contributed to this rise in heat fluxes.

To assess the influence of the MHW on heat fluxes, we compared the CTL run with the CLM run (Fig. 4b). The variation in the heat fluxes between the two runs exceeded 200 W/m^2^ in parts of the KE-LM region. The spatial distribution of these differences corresponds to the SST differences illustrated in Fig. 1b and Supplementary Fig. 1, suggesting that the MHW impacts atmospheric conditions by elevating surface heat fluxes.

To further verify the effect of the MHW on the near-surface atmospheric thermodynamic field, Fig. 4c illustrates the difference in equivalent potential temperature ($\:{\theta\:}_{e}$) at 500 m altitude between the CTL and CLM runs. This difference exceeds 4 K over the ocean east of Chiba Prefecture, with the peak $\:{\theta\:}_{e}$ difference located south of the peak heat flux difference (Fig. 4b and c). This displacement of peaks is likely due to the advection by synoptic-scale northerly winds. More than 70% of the $\:{\theta\:}_{e}$ difference is due to the water vapor difference (Supplementary Fig. 3).

Subsequently, we investigated the impact of MHW on the atmospheric dynamical field. Figure 4d presents the wind speed differences between the CTL and CLM runs at 500 m altitude. The differences are less than 1 m/s. Although it is commonly reported that SST anomalies in mid-latitudes can alter the dynamical field^12^, the present findings suggest that the effect is minimal in this case.

### Effects of the marine heatwave on atmospheric conditions during the extreme precipitation

This section analyzes atmospheric conditions between 01:00 and 08:00 JST on September 8, 2023, during which the precipitation disparity between the CTL and CLM runs expanded in eastern Chiba Prefecture (Fig. 3d). The conditions conducive to precipitation enhancement differed between the early half (01:00–04:00 JST) and the latter half (05:00–08:00 JST), as detailed in the subsequent paragraphs. Accordingly, the conditions in the early and later halves were analyzed. The total precipitation difference between the two runs during this time was 212 mm (Fig. 3d), with contributions of 43.4% (92 mm) from the early half and 56.6% (120 mm) from the latter half. Thus, both periods are crucial for assessing the impact of the MHW on the heavy precipitation.

**Fig. 5:**
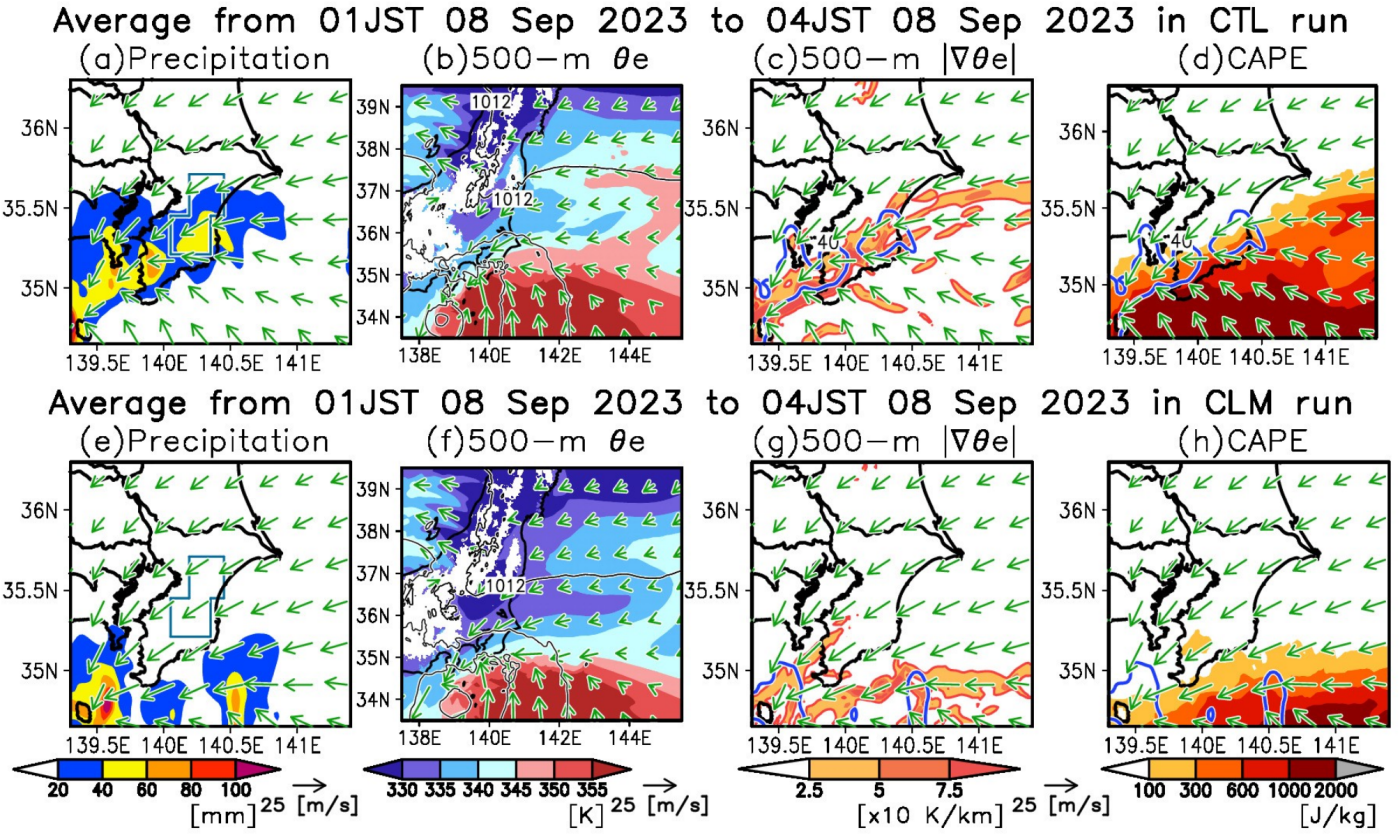
Effects of the marine heatwave on atmospheric conditions during the early half of the extreme precipitation. (**a**) Horizontal distributions of surface precipitation (shading) and horizontal wind vectors at 500 m altitude (arrows) averaged during the early half of the heavy precipitation (01:00–04:00 JST on September 8, 2023) in the CTL run. A blue line is same as that depicted in Fig. 3c. (**b**) Same as (**a**), but the shading indicates $\:{\theta\:}_{e}$ at 500 m altitude. Black contours exhibit SLP at 4 hPa intervals. (**c**) Same as (**a**), but the shading indicates the magnitude of the horizontal gradient of $\:{\theta\:}_{e}$ at 500 m altitude. Blue contours portray the surface precipitation of 40 mm. (**d**) Same as (**c**), but the shading indicates convective available potential energy (CAPE). We calculated the CAPE by lifting an air parcel located at the height where $\:{\theta\:}_{e}$ reaches its maximum at each grid. (**e–h**) Same as (**a–d**), but for the CLM run.

Initially, we examine the CTL runs during the early half of the period (Fig. 5a–d). During this phase, easterly winds from the KE-LM region directed toward the heavy precipitation area in eastern Chiba Prefecture near the surface (Fig. 5a). Near the coast of eastern Chiba Prefecture, a pronounced east–west contrast in equivalent potential temperature ($\:{\theta\:}_{e}$) at 500 m altitude is observed (Fig. 5b), and a surface front characterized by steep horizontal $\:{\theta\:}_{e}$ gradients forms (Fig. 5c). Heavy precipitation occurs over this surface front (Fig. 5c). Such thermodynamical conditions are also seen in ERA5 (Supplementary Fig. 4a, b). Over the ocean upstream of this front, the Convective Available Potential Energy (CAPE) exceeds 300 J/kg (Fig. 5d). This suggests that the instability intensifies over the ocean and then is released over the front, which enhances precipitation during the early half.

Subsequently, we analyze the CLM run during the same phase (Fig. 5e–h). In the CLM run, precipitation is absent over the eastern part of Chiba Prefecture (Fig. 5e). Compared to the CTL run, the $\:{\theta\:}_{e}$ at 500 m altitude over the ocean east of Chiba Prefecture is lower (Fig. 5f), reflecting the influence of the warm SST anomalies associated with the MHW (refer to Fig. 4c). Additionally, the thermal contrast between the land and ocean around eastern Chiba Prefecture is less pronounced in the CLM run than in the CTL run (Fig. 5f), and no fronts are evident (Fig. 5g). Over the ocean upstream of Chiba Prefecture, CAPE is below 100 J/kg, indicating a stable atmospheric condition in the CLM run (Fig. 5h). These findings indicate that the KE-LM-induced MHW contributes to the precipitation enhancement during the early half through the formation of the surface front and the enhancement of the atmospheric instability.

**Fig. 6:**
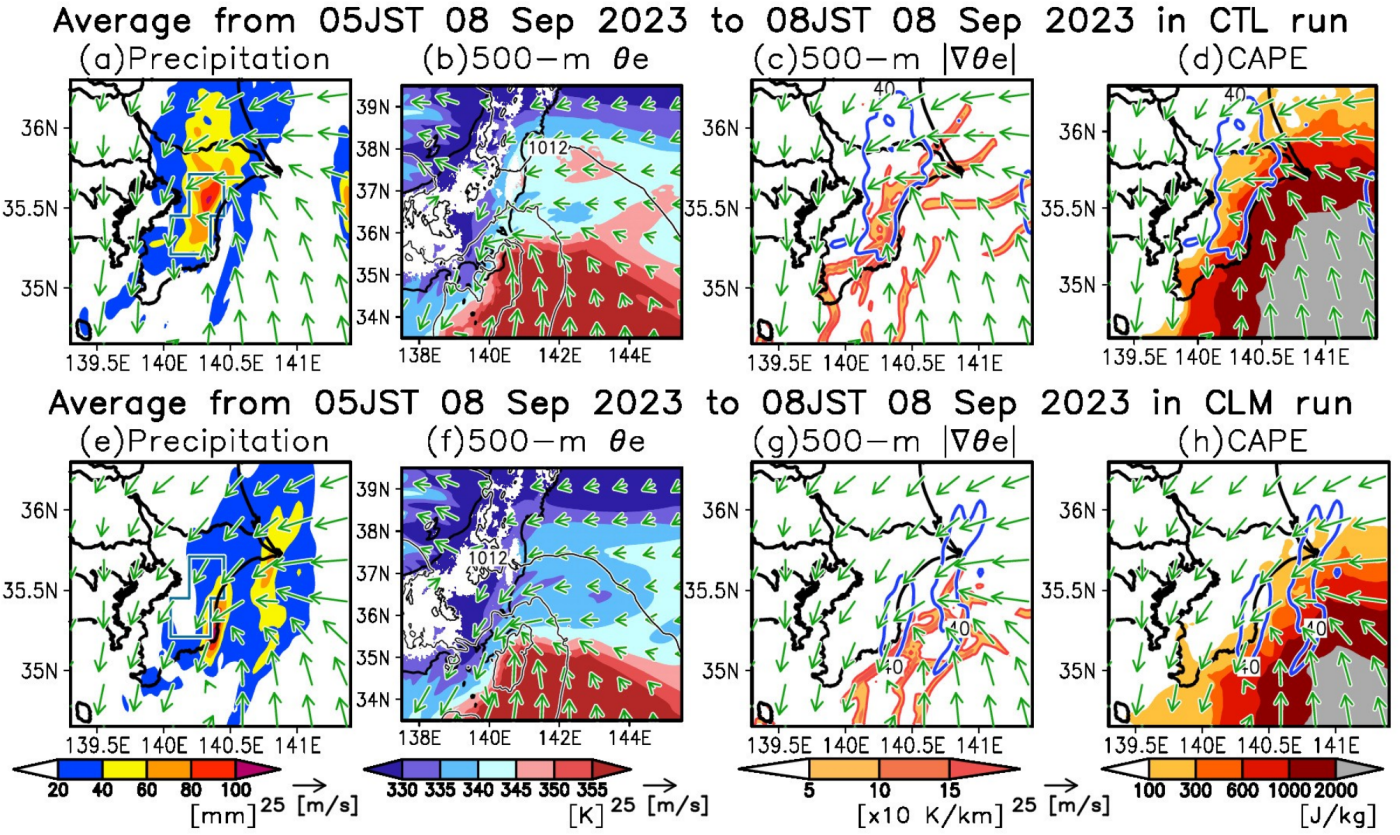
Effects of the marine heatwave on atmospheric conditions during the latter half of the extreme precipitation. Same as Fig. 5, but for the latter half of the heavy precipitation (05:00–08:00 JST on September 8, 2023).

Next, we examined the CTL run during the latter half of the heavy precipitation (Fig. 6a–d). During this phase, southeasterly winds transported high $\:{\theta\:}_{e}$ air into the precipitation zone over eastern Chiba Prefecture (Fig. 6a). A front developed between the higher $\:{\theta\:}_{e}$ (> 345 K) associated with the southeasterly winds over the ocean and the lower $\:{\theta\:}_{e}$ (< 345 K) associated with the northeasterly winds over the land (Fig. 6b, c). The $\:{\theta\:}_{e}$ features similar with the CTL run can be observed in ERA5 (Supplementary Fig. 4c, d). In the region southeast of the precipitation area, the CAPE exceeded 1000 J/kg (Fig. 6d). The release of this substantial CAPE at the front appears to contribute to the intensified precipitation observed during the latter half. Compared to the early half, the CAPE upstream of the precipitation zone was greater in the latter half, explaining the increased precipitation intensity.

Furthermore, we analyzed the CLM run during the latter half (Fig. 6e–h). Relative to the CTL run, the precipitation zone in the CLM run shifted eastward towards the sea (Fig. 6e). However, the precipitation intensity in the CLM run was generally similar to that in the CTL run (Fig. 6a, e). The CAPE upstream of the precipitation zone in the CLM run also paralleled that in the CTL run (Fig. 6d, h), corresponding to the comparable precipitation intensity between the two runs. The air associated with high CAPE appeared to flow from the oceanic area south of the KE-LM region (Fig. 6d, h). Consequently, the MHW associated with the KE-LM did not influence the atmospheric instability that led to heavy precipitation in the latter half. However, a notable difference was observed in the position of the surface front associated with convergence line between the CTL and CLM runs (Fig. 6c, g). The surface front associated with the convergence in the CLM run was positioned further seaward compared to the CTL run, with intense precipitation occurring along this front over the ocean. Therefore, the shift in the position of the front accounts for the differences in precipitation between the CTL and CLM runs during the latter half.

**Fig. 7:**
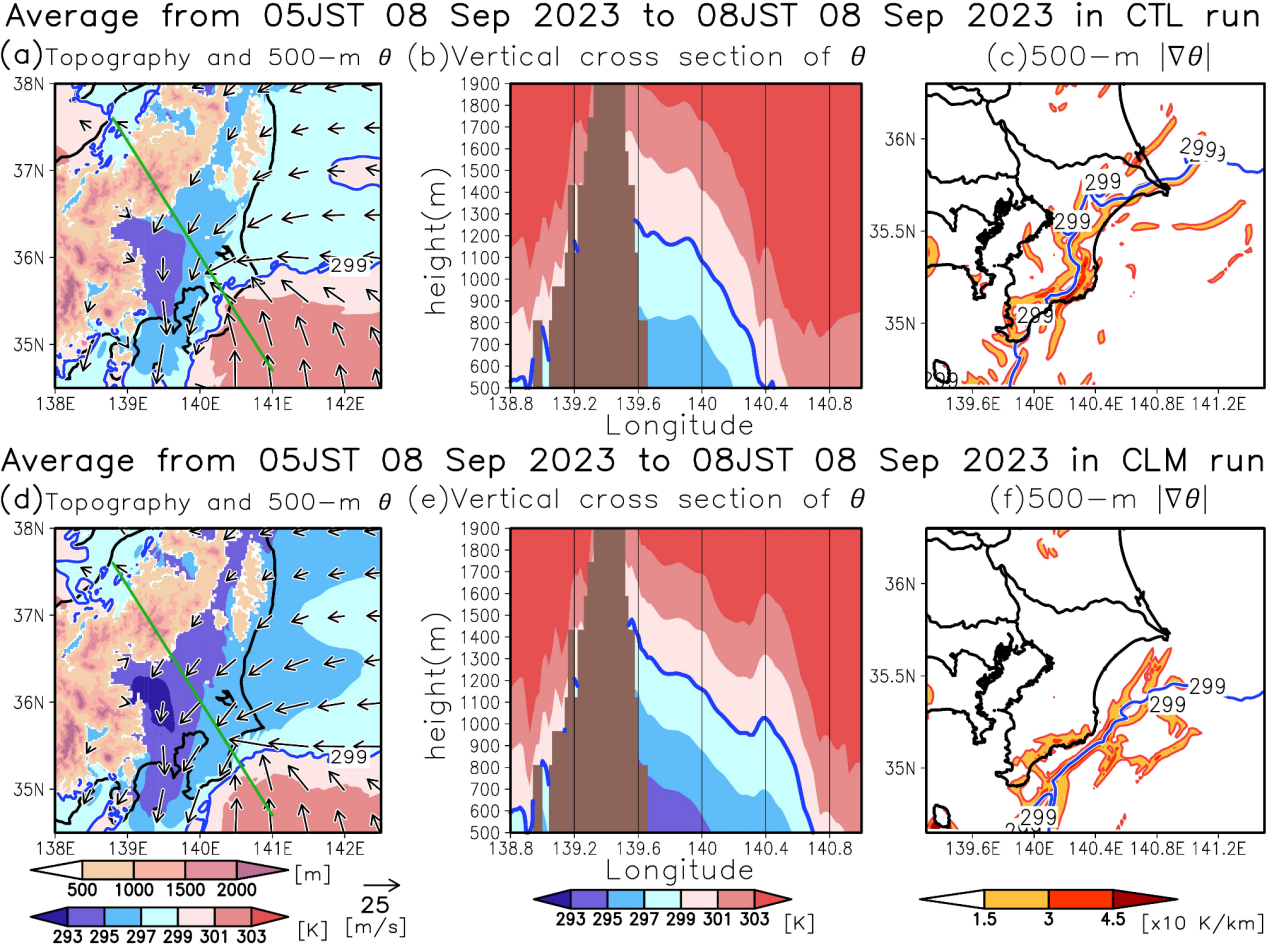
Effects of the marine heatwave on the cold air over land and the position of the front during the latter half of the extreme precipitation. (**a**) Same as Fig. 6a but for shading range from blue to red indicates potential temperature ($\:\theta\:$) at 500 m altitude, and brown shading indicates topography in the CTL run. A blue line indicates the isentropic line of 299 K. (**b**) Cross sectional map of $\:\theta\:$ along the green line in (**a**). A blue line indicates the isentropic line of 299 K. (**c**) Same as (**a**), but the shading indicates the magnitude of the horizontal gradient of $\:\theta\:$ at 500 m altitude. (**d–f**) Same as (**a–c**), but for the CLM run.

Why, then, did the position of the front shift seaward in the CLM run compared to the CTL run during the latter period? This shift can be attributed to differences in the cold air on the landward side of the surface front (Fig. 7). The interior of Chiba Prefecture is encircled by mountains exceeding 1,500 m in elevation (Fig. 7a, b, d, e), which are known to trap cold air^13,14^, a phenomenon similar to cold air damming observed in the Northeastern United States^15,16^. The pattern of horizontal wind vectors at 500 m altitude indicates that air on the landward side of the front originated from the KE-LM region (Fig. 7a, d). Consequently, the near-surface potential temperatures ($\:\theta\:$) on the landward side of the front are lower in the CLM run (Fig. 7d, e) than in the CTL run (Fig. 7a, b). Conversely, the near-surface $\:\theta\:$ on the warm side of the front is similar in both runs (Fig. 7a, d), as noted previously. To examine the impact of cold air on the front’s location, we analyzed the 299-K isentropic line, which aligns with the steep horizontal $\:\theta\:$ gradient (Fig. 7c, f) and the convergence line (Fig. 7a, d) at 500 m altitude in both runs. In the CLM run, this isentropic line is positioned seaward compared to the CTL run owing to the relatively colder air on the land side (Fig. 7a–f). Additionally, the synoptic-scale atmospheric circulation associated with the typhoon was almost the same in the two runs (Supplementary Fig. 5). These findings indicate that the MHW associated with the KE-LM leads to warmer air on the landward side of the front, facilitating the front’s development over land and contributing to the heavy precipitation over eastern Chiba Prefecture during the latter period.

## Discussion

The recent MHW caused by the unprecedented KE-LM may contribute to heavy precipitation in Japan, although this has not yet been extensively studied. To clarify this possible influence of the MHW, this study examines the record-breaking precipitation event that occurred in the eastern part of Chiba Prefecture, Japan, on September 8, 2023, using cloud-resolving numerical experiments. The results indicate that the warm SST anomalies associated with the MHW potentially increased precipitation by approximately 300 mm, accounting for ~ 70% of the total precipitation observed in the CTL run (Fig. 3). Moreover, this study explores how the MHW enhances the precipitation. It reveals that the MHW modifies the near-surface atmosphere by increasing surface heat fluxes (Fig. 4). In the early phase of the heavy precipitation, the MHW contributed to precipitation enhancement through the formation of a surface front and increased atmospheric instability (Fig. 5). In the latter phase, the MHW influenced the position of a front by warming the cold air on the landward side, leading to further precipitation enhancement over eastern Chiba Prefecture (Figs. 6 and 7). Thus, the KE-LM-associated MHW significantly contributed to the occurrence of the extreme precipitation in September 2023 by enhancing atmospheric instability and affecting the formation and positioning of surface fronts. This study is the first to demonstrate the influence of the recent MHW caused by the unprecedented KE-LM on extreme precipitation events.

This research shows that the MHW enables the development of surface fronts over land (Figs. 6 and 7), suggesting that the KE-LM-induced MHW increases the risk of frontal heavy precipitation over the land around the east coast of Japan, with societal impacts. In Chiba Prefecture, costal fronts, such as the fronts investigated in this study, frequently occur and are associated with heavy precipitation^13,17^. The effect of the MHW on the location of the front observed in this study may also be relevant to other extreme weather events. Future research will statistically investigate this issue using observational and reanalysis data. Furthermore, similar coastal fronts occur over the Northeastern United States^18,19^, and recent years have seen MHWs off the east coast of the United States^20–22^. Therefore, the effects of MHWs identified in this study may not be limited to Japan but could also be relevant to the Northeastern United States, which warrants further investigation.

The variability in the western boundary currents, such as the KE discussed in this study, leads to significant SST warming^1,23,24^. Additionally, previous research has demonstrated that the long-term warming trend of SST is more pronounced over the western boundary currents in all ocean basins than in other regions^25–27^. Consequently, MHWs are more likely to occur in these areas due to multiple warming factors. Moreover, this study suggests that MHWs may increase the risk of extreme weather events. Therefore, a deeper understanding of variability of the western boundary currents, their connection to global warming, and their impact on extreme weather events is crucial for comprehending coastal climates worldwide and for accurately assessing future climate projections.

A recent study that examined the influence of external forcing and internal variability on the increasing trend in the frequency of global marine heatwaves during 1982–2021, could show that the external forcing significantly contributes to the trend by warming the mean SST^28^. To verify the change in mean SST in the KE-LM region (indicated by a green line in Fig. 1b), we examined the long-term trends in the SST using three analysis data (Supplementary Fig. 6). The results show that the SST in September 2023 is much warmer than the value estimated from the long-term trend. This strongly suggests that the warm SST anomalies highlighted in this study are mainly caused by the KE-LM. It is important to note that the climatological values used in this study (1985–2012 average) already include the influence of climate change (Supplementary Fig. 6).

Furthermore, it is known that the Pacific Decadal Oscillation (PDO) also fluctuates SST to the east of Japan by changing the path state of the KE^1,2,29^. The phase of the PDO shifted from positive to negative after 2018, and it was negative in 2023^2^. This negative PDO since 2018 stabilizes the KE state^2^. During the stable state of the KE, the KE generally flows straight to the east, leading to cold SST anomalies over the KE-LM region^30–32^. However, in 2023, the KE meandered northward, which caused significant warm SST anomalies. As mentioned in the introduction, this meandering of the KE has been observed for the first time since satellite measurements of SSH began. Thus, the SST anomaly in the KE-LM region in September 2023 is different from the well-known feature related to the PDO. The mechanism and reason behind the occurrence of the meandering of the KE are currently under discussion, and further research on this topic is needed.

## Methods

### Data

The SSH and surface geostrophic ocean current shown in Fig. 1a are based on the global ocean gridded L4 data provided by the Copernicus Marine Environment Monitoring Service (https://data.marine.copernicus.eu/product/SEALEVEL_GLO_PHY_CLIMATE_L4_MY_008_057/description). The SST data for Fig. 1b and the numerical experiments are from the Coral Reef Watch (CRW) v3.1 daily global 5 km SST dataset produced by the National Oceanic and Atmospheric Administration (NOAA)^33,34^. The Marine Heatwave Watch v1.0.1 product produced by the NOAA is used to identify areas experiencing MHWs in Fig. 1b. This product is created by applying the MHW algorithm of ref. 35 to the CRW v3.1 SST data, and the climatology used to define MHWs in this product is average from 1985 to 2012 (see https://coralreefwatch.noaa.gov/product/marine_heatwave/ for the details). This climatology is also used as the baseline for calculating SST anomalies in this study. The observational precipitation data used in Figs. 1d and e and 2 are the Radar/Raingauge-Analyzed Precipitation data produced by the JMA (https://www.jma.go.jp/jma/en/Activities/qmws_2018/Presentation/3.1/Radar%20Rain%20Gauge-Analyzed%20Precipitation_revised.pdf) and the on-site observational data at JMA stations (https://www.jma.go.jp/jma/en/Activities/amedas/amedas.html). Figure 1f is created using ERA5 data^36^. For the atmospheric initial and boundary conditions in the numerical experiments, analysis data from the JMA Global Spectral Model^37^ was employed. Extended Reconstructed Sea Surface Temperature (ERSST) Level 4 version 5^38^, Hadley Centre Sea Ice and SST (HadISST) version 1^39^, and COBE SST version 2^40^ are used to create Supplementary Fig. 6.

### Numerical experiments

The Cloud Resolving Storm Simulator (CReSS)^11^ was utilized for the numerical experiments in this study. The model included parameterization of cloud microphysical processes and subgrid-scale turbulence using a cold rain scheme^41^ and a 1.5-order turbulent kinetic energy closure model^42^, respectively. This setup is similar to that reported in Ref. 9. The horizontal grid spacing of the model was set to 0.02° in both latitudinal and longitudinal directions. The model atmosphere comprised 48 vertical layers, with the lowest layer being 100 m thick and the top altitude at 19,200 m. The model domain spanned from 130.01–155.93°E and from 30.01–49.19°N. The initial time for the experiments was set to 21:00 JST on September 6, 2023, approximately 1.1 days before the onset of the heavy precipitation event at 00:00 JST on September 8, 2023.

In the described experimental setup, the CTL and CLM runs were performed using distinct SST patterns. For the CTL run, the SST pattern corresponding to the experiment’s initial day was used. Conversely, in the CLM run, the SST pattern was substituted with climatological data within the KE-LM region, which is delineated by the green line in Fig. 1b. The spatial distribution of SST in both runs and their differences are depicted in Supplementary Fig. 1. Discontinuity in the SST is observed at the boundary of the modified region in the CLM run; however, this discontinuity does not significantly affect the precipitation or the dynamic and thermodynamic structures of the near-surface atmosphere at the boundary, as shown in Supplementary Fig. 7.

Additionally, nine SST patterns were generated by linearly reducing the SST within the KE-LM region (marked by a green line in Fig. 1b) in increments of 10% from the value used in the CTL run to that used in the CLM run. Nine further numerical experiments were conducted using these SST patterns. The outcomes from these additional experiments, along with those from the CTL and CLM runs, are presented in Supplementary Fig. 2.

## Electronic supplementary material

Below is the link to the electronic supplementary material.

Supplementary Material 1

## Data Availability

The SSH and surface geostrophic ocean current data used during the current study are available in the Copernicus Marine Environment Monitoring Service website, [https://data.marine.copernicus.eu/product/SEALEVEL_GLO_PHY_CLIMATE_L4_MY_008_057/description]. The CRW SST data used during the current study are available in NOAA website, [https://www.star.nesdis.noaa.gov/pub/sod/mecb/crw/data/5km/v3.1_op/]. The MHW data used during the current study are available in NOAA website, [https://www.star.nesdis.noaa.gov/pub/sod/mecb/crw/data/marine_heatwave/v1.0.1/]. JMA surface observational data used during the current study are available in the JMA website, [https://www.data.jma.go.jp/stats/etrn/index.php]. The Radar/Raingauge-Analyzed Precipitation dataset used during the current study are available in the Japan Meteorological Business Support Center, [http://www.jmbsc.or.jp/en/index-e.html]. ERA5 used during the current study are available in Copernicus Climate Data Store, [https://cds.climate.copernicus.eu/]. The JMA Global Spectral Model data used during the current study are available in the website of the Research Institute for Sustainable Humanosphere, Kyoto University, [http://database.rish.kyoto-u.ac.jp/index-e.html]. ERSST version 5 are available in NASA’s Physical Oceanography Distributed Active Archive Center, [https://podaac.jpl.nasa.gov/dataset/REYNOLDS_NCDC_L4_MONTHLY_V5]. HadISST version 1 are available in Met Office website, [https://www.metoffice.gov.uk/hadobs/hadisst/data/download.html]. COBE SST version 2 are available in NOAA website, [https://psl.noaa.gov/data/gridded/data.cobe2.html]. The numerical experiment datasets analyzed during the current study are available from the corresponding author on reasonable request.

## Code availability

The Code of CReSS model can be downloaded from https://github.com/cress-nagoya/CReSS. All figures in this paper were generated with the Grid Analysis and Display System (GrADS) version 2.0.2, an open source software distributed by the Center for Ocean-Land-Atmosphere Studies (http://cola.gmu.edu/grads). The codes used in this study are available upon request from the corresponding author.
