# Supplementary material for: Effects of a marine heatwave associated with the Kuroshio Extension large meander on extreme precipitation in September 2023
Source: Sci Rep. 2025 Feb 13;15:5332. doi: 10.1038/s41598-025-88294-9 (PMC11825860; doi:10.1038/s41598-025-88294-9)
Supplement: Supplementary file 1 — Supplementary Material 1 [file 41598_2025_88294_MOESM1_ESM.pdf]

Supplementary Information

Effects of a marine heatwave associated with the Kuroshio Extension large meander on extreme precipitation in September 2023

Hidetaka Hirata[1], Ryuichi Kawamura[2], and Masami Nonaka[3]

[1] *Department of Data Science, Faculty of Data Science, Rissho University, Kumagaya, Japan*

[2] *Department of Earth and Planetary Sciences, Faculty of Science, Kyushu University, Fukuoka, Japan*

[3] *Application Laboratory, Japan Agency for Marine-Earth Science and Technology, Yokohama, Japan*

Corresponding author: Hidetaka Hirata (hirata@ris.ac.jp)

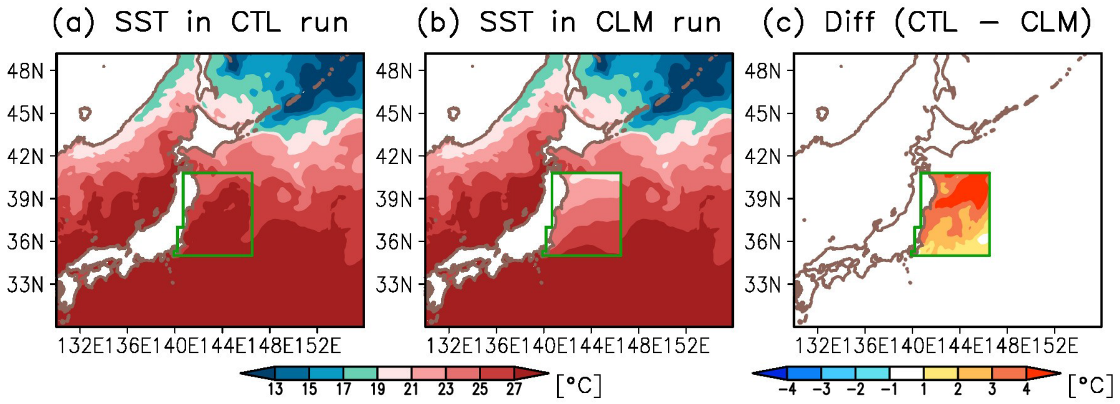

Supplementary Figure 1. Sea surface temperature in the numerical experiments.

a Horizontal distributions of sea surface temperature (SST) in the control (CTL) run. The ocean area enclosed by the green line corresponds to the area where the SST is replaced by the climatology in the climate (CLM) run. **b** Same as (**a**), but for the CLM run. **c** Horizontal distribution of the SST difference between the CTL and CLM runs.

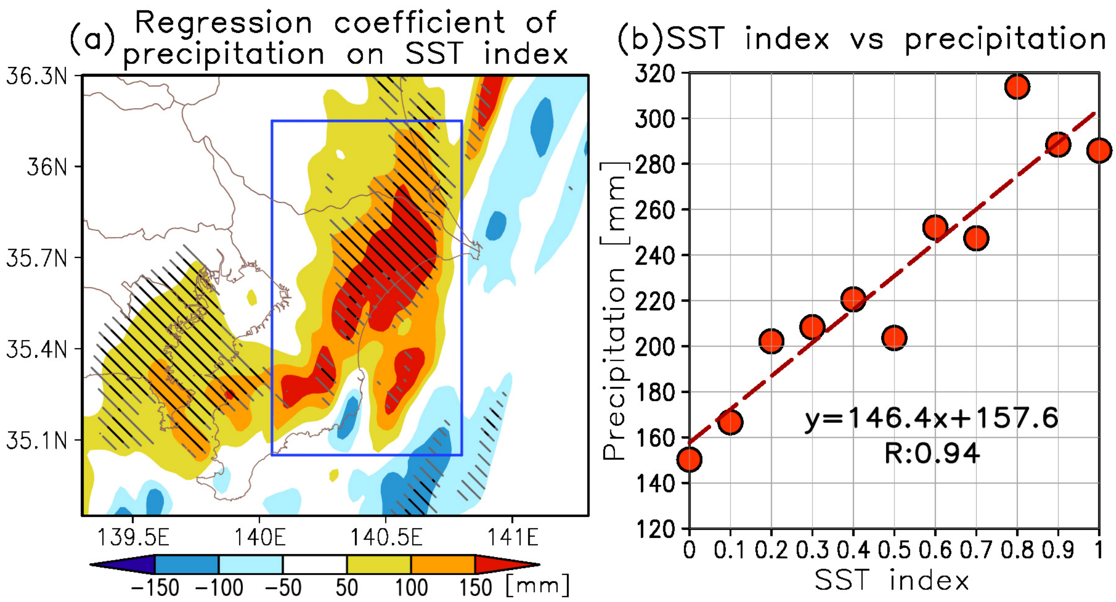

Supplementary Figure 2. Linear regression of precipitation on SST anomalies within the Kuroshio Extension Large meandering region using the data of 11 numerical experiments. **a** Regression coefficient of precipitation accumulated from 00:00–13:00 JST on September 8, 2023, on the SST indices estimated from the data of 11 numerical experiments (see “Methods” in the main manuscript for details of the design of the experiments). The SST indices in each experiment are defined by normalizing the area-averaged SST anomaly within the green line in Supplementary Fig. 1 with the area-averaged SST anomaly in the CTL run (i.e., the SST index for the CTL and CLM runs is 1.0 and 0.0, respectively, and the index for the other experiments varies linearly between them). Black and gray diagonal lines indicate statistical significance at the 95% and 90% confidence levels based on a two-tailed Student's t-test. **b** Scatter plots between the SST indices and the precipitation which is calculated by averaging the accumulated precipitation on the grids with the regression coefficients greater than100 mm within the blue box in (**a**). Linear regression equation and correlation coefficient (R) obtained from these data are also shown.

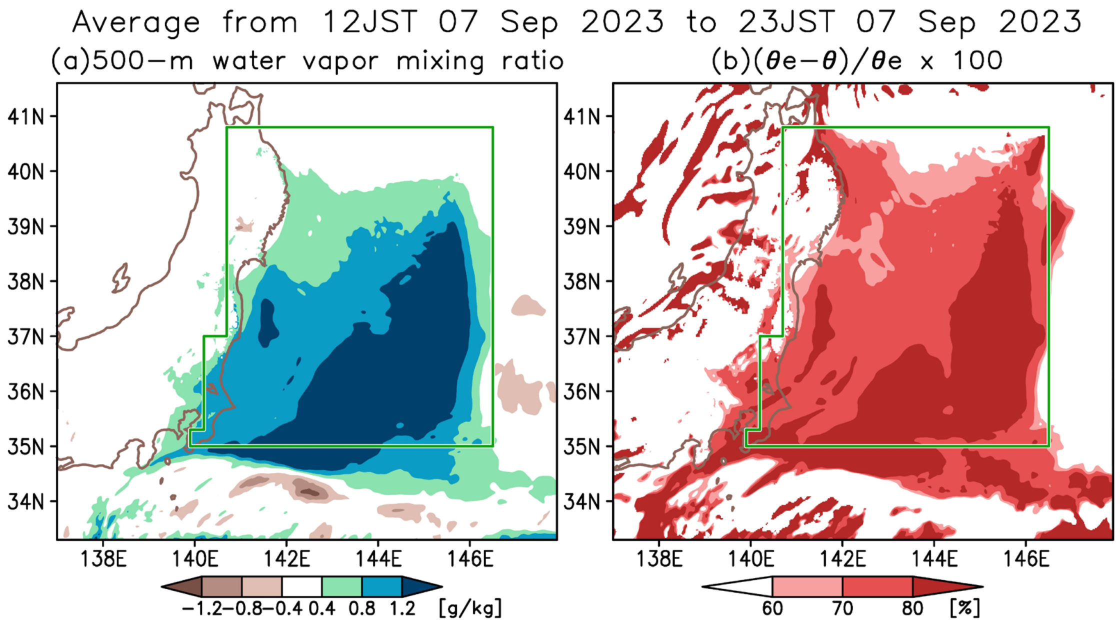

Supplementary Figure 3. Effects of the marine heatwave on atmospheric conditions prior to the extreme precipitation.

a Horizontal distribution of the difference between the CTL and CLM runs for the water vapor mixing ratio at 500 m altitude averaged from 12:00–23:00 JST on 7 September 2023. **b** Horizontal distribution of the ratio of the differences between equivalent potential temperature (θ_e) and potential temperature (θ) to θ_e at 500 m altitude. To calculate the ratio, we used the difference between the CTL and CLM runs for θ_e and θ averaged from 12:00–23:00 JST on 7 September 2023. The ratio where the θ_e differences are less than 0 K is suppressed.

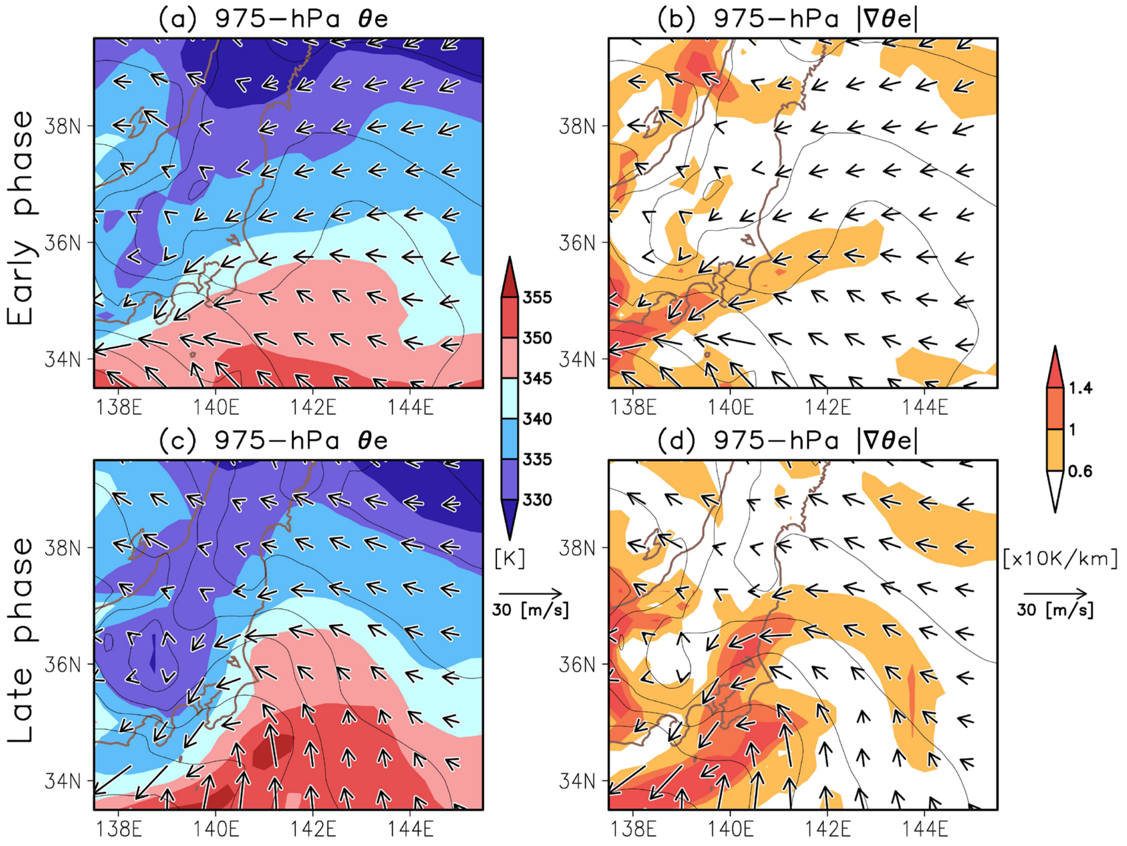

Supplementary Figure 4. Atmospheric conditions during the heavy precipitation in reanalysis data

a Horizontal distribution of θ_e at 975 hPa (shading), horizontal wind vectors at 975 hPa (arrows), and sea level pressure (contour) averaged during the early phase of the heavy precipitation (03:00–06:00 JST on September 8, 2023) in the ERA 5 reanalysis. **b** Same as (a), but the shading indicates the magnitude of the horizontal gradient of θ_e at 975 hPa. **c–d** Same as (**a–b**), but for the late phase of the heavy precipitation (09:00–12:00 JST on September 8, 2023).

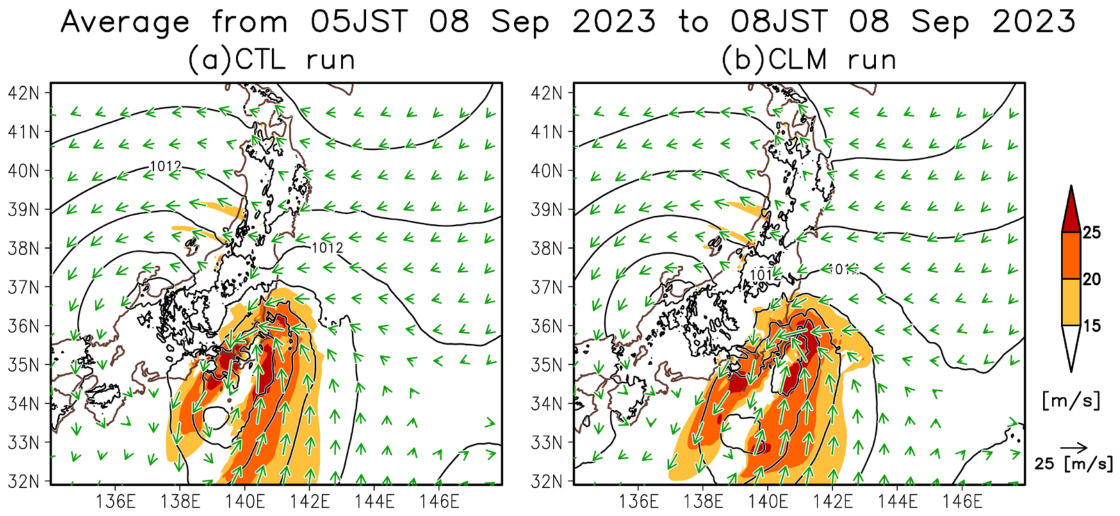

Supplementary Figure 5. Synoptic-scale atmospheric conditions during the latter half of the extreme precipitation.

a Horizontal distribution of magnitude of horizontal wind at 500 m altitude (shading) and horizontal wind vectors at 500 m altitude (arrows) averaged during the latter half of the heavy precipitation (05:00–08:00 JST on September 8, 2023) in the CTL run. Black contours exhibit sea level pressure at 2 hPa intervals. **b** Same as (**a**), but for the CLM run.

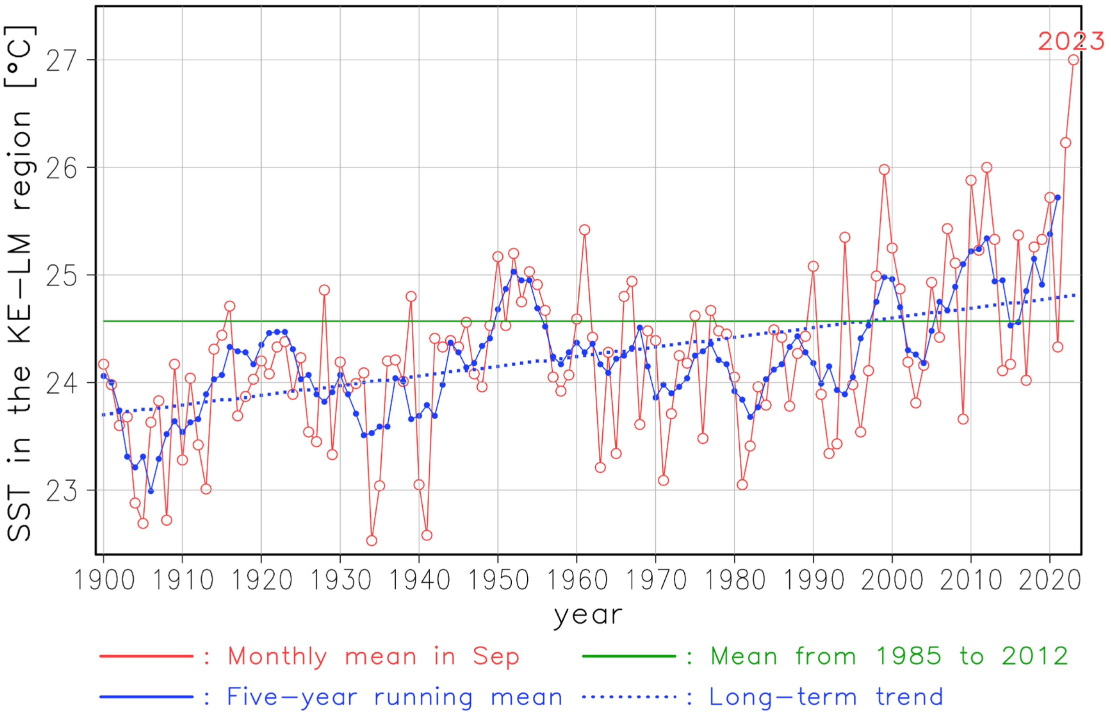

Supplementary Figure 6. Long-term variability and trend of SST in the Kuroshio Extension large meander region

Time series of SST averaged over the oceanic area enclosed by the green line in Fig. 1a from 1990 to 2023. Red and blue solid lines display the monthly mean for September and its five-year running mean, respectively. The dashed blue line indicates the long-term trend estimated from the five-year running mean. The green sold line indicates the mean value from 1985–2012, corresponding to the climatology used in this study. The SST values shown are averages of three analysis data sets: Extended Reconstructed Sea Surface Temperature (ERSST) Level 4 version 5, Hadley Centre Sea Ice and SST (HadISST) version 1, and COBE SST version 2.

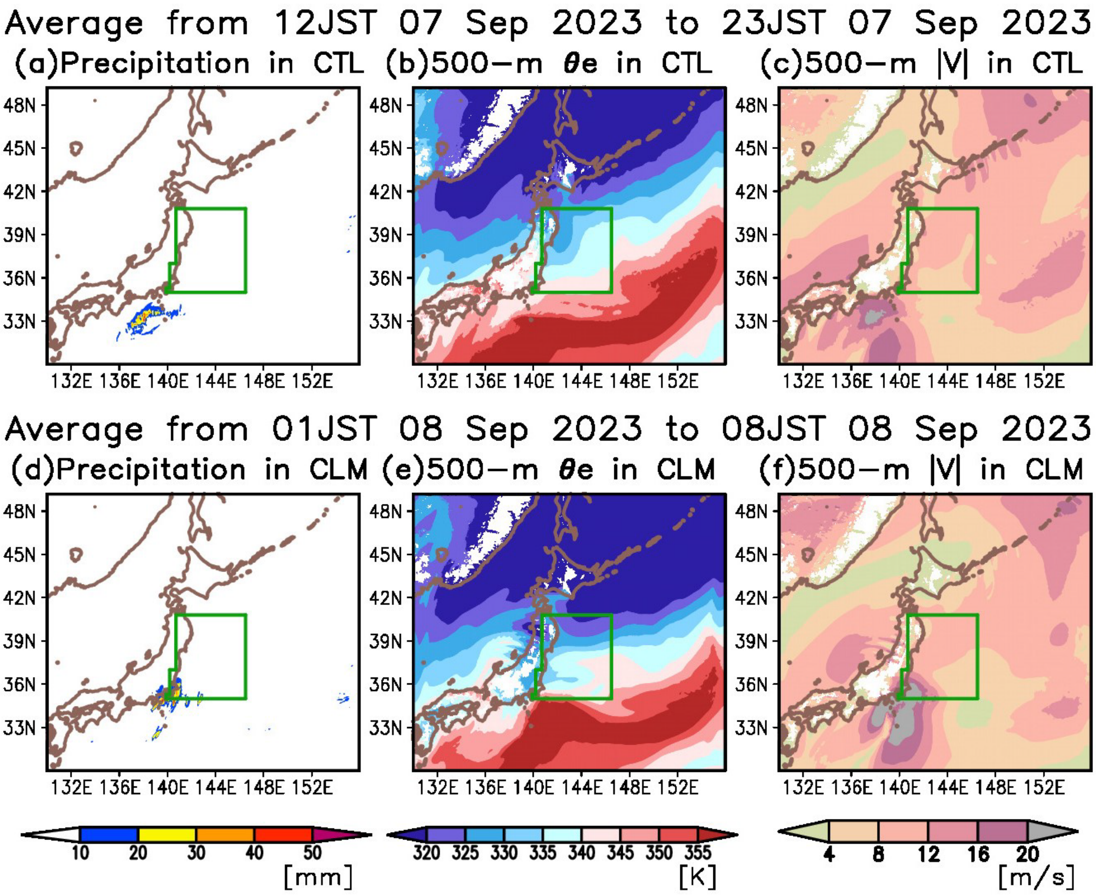

Supplementary Figure 7. Precipitation and the dynamic and thermodynamic structure of the near-surface atmosphere in the CLM run.

a Horizontal distributions of surface precipitation (shading) averaged from 12:00–23:00 JST on September 7, 2023, in the CLM run. The ocean area enclosed by the green line corresponds to the area where the SST is modified in the CLM run. **b** Same as (**a**), but the shading indicates θ_e at 500 m altitude. **c** Same as (**a**), but the shading indicates the magnitude of horizontal wind speed at 500 m altitude. **d**–**f** Same as (**a**–**c**), but for the average from 01:00–08:00 JST on September 8, 2023, in the CLM run.
